# A personalized prediction model for urinary tract infections in type 2 diabetes mellitus using machine learning

**DOI:** 10.3389/fphar.2023.1259596

**Published:** 2024-01-05

**Authors:** Yu Xiong, Yu-Meng Liu, Jia-Qiang Hu, Bao-Qiang Zhu, Yuan-Kui Wei, Yan Yang, Xing-Wei Wu, En-Wu Long

**Affiliations:** ^1^ Institute of Materia Medica, Chinese Academy of Medical Sciences and Peking Union Medical College, Beijing, China; ^2^ Department of Pharmacy, Daping Hospital, Army Medical University, Chongqing, China; ^3^ Personalized Drug Therapy Key Laboratory of Sichuan Province, Department of Pharmacy, Sichuan Provincial People’s Hospital, School of Medicine, University of Electronic Science and Technology of China, Chengdu, Sichuan, China; ^4^ School of Pharmacy, Southwest Medical University, Luzhou, Sichuan, China; ^5^ Department of Endocrinology and Metabolism, Sichuan Provincial People’s Hospital, University of Electronic Science and Technology of China, Chengdu, Sichuan, China; ^6^ Chinese Academy of Sciences Sichuan Translational Medicine Research Hospital, Chengdu, Sichuan, China

**Keywords:** type 2 diabetes mellitus, urinary tract infections, machine learning, predictive models, individualized therapy

## Abstract

Patients with type 2 diabetes mellitus (T2DM) are at higher risk for urinary tract infections (UTIs), which greatly impacts their quality of life. Developing a risk prediction model to identify high-risk patients for UTIs in those with T2DM and assisting clinical decision-making can help reduce the incidence of UTIs in T2DM patients. To construct the predictive model, potential relevant variables were first selected from the reference literature, and then data was extracted from the Hospital Information System (HIS) of the Sichuan Academy of Medical Sciences and Sichuan Provincial People’s Hospital for analysis. The data set was split into a training set and a test set in an 8:2 ratio. To handle the data and establish risk warning models, four imputation methods, four balancing methods, three feature screening methods, and eighteen machine learning algorithms were employed. A 10-fold cross-validation technique was applied to internally validate the training set, while the bootstrap method was used for external validation in the test set. The area under the receiver operating characteristic curve (AUC) and decision curve analysis (DCA) were used to evaluate the performance of the models. The contributions of features were interpreted using the SHapley Additive ExPlanation (SHAP) approach. And a web-based prediction platform for UTIs in T2DM was constructed by Flask framework. Finally, 106 variables were identified for analysis from a total of 119 literature sources, and 1340 patients were included in the study. After comprehensive data preprocessing, a total of 48 datasets were generated, and 864 risk warning models were constructed based on various balancing methods, feature selection techniques, and a range of machine learning algorithms. The receiver operating characteristic (ROC) curves were used to assess the performances of these models, and the best model achieved an impressive AUC of 0.9789 upon external validation. Notably, the most critical factors contributing to UTIs in T2DM patients were found to be UTIs-related inflammatory markers, medication use, mainly SGLT2 inhibitors, severity of comorbidities, blood routine indicators, as well as other factors such as length of hospital stay and estimated glomerular filtration rate (eGFR). Furthermore, the SHAP method was utilized to interpret the contribution of each feature to the model. And based on the optimal predictive model a user-friendly prediction platform for UTIs in T2DM was built to assist clinicians in making clinical decisions. The machine learning model-based prediction system developed in this study exhibited favorable predictive ability and promising clinical utility. The web-based prediction platform, combined with the professional judgment of clinicians, can assist to make better clinical decisions.

## 1 Introduction

Diabetes Mellitus (DM) is a heterogeneous group of metabolic disorders characterized by chronic hyperglycemia that arises from defects in insulin secretion, insulin action, or both (American Diabetes Association, 2014). According to the statistical report by the IDF (International Diabetes Federation), the number of adult patients with DM worldwide has reached 537 million in 2021. The prevalence of DM is increasing annually with an average growth rate of approximately 56%. It is estimated that by 2045, the global burden of DM will reach 783 million (Dorresteijn et al., 2011). Notably, China remains the country with the highest number of individuals affected by DM, with 140.9 million and 174.4 million people in 2021 and 2045, respectively (Dorresteijn et al., 2011). Type 2 diabetes mellitus (T2DM) accounts for the vast majority (over 90%) of diabetes worldwide (Dorresteijn et al., 2011). T2DM can result in a broad spectrum of health complications and organ damage (Gregg et al., 2016), including cardiovascular diseases (Rawshani et al., 2018), neuropathy (Davies et al., 2006), retinopathy (Tan et al., 2017), nephropathy (Ritz and Orth, 1999), foot ulcers and amputations (Liu et al., 2015a). In addition, T2DM is associated with an increased risk of infectious disease (Shah and Hux, 2003). The increased susceptibility of individuals with T2DM to infectious diseases can be attributed to multiple factors, including immune dysfunction (Wang et al., 2022), impaired wound healing (Kimball et al., 2018; Xiong et al., 2020), and a higher prevalence of comorbidities such as obesity and cardiovascular disease (Piché et al., 2020; Wu and Ballantyne, 2020). Hyperglycemia in T2DM patients may further compromise immune function, creating a conducive environment for bacterial and viral growth. Consequently, the risk of various infections, such as respiratory infections, skin infections, hyperglycemia and urinary tract infections (UTIs) is heightened among individuals with T2DM [(Muller et al., 2005; Lalla and Papapanou, 2011; Carrillo-Larco et al., 2022)].So the association between UTIs and T2DM has been well established (Flores-Mireles et al., 2015).

UTIs is an infection of the urinary system, caused by a range of pathogens, but most commonly by *Escherichia coli, Klebsiella pneumoniae, Proteus mirabilis, Enterococcus faecalis and Staphylococcus saprophyticus* (Flores-Mireles et al., 2015).UTIs are some of the most common bacterial infections, affecting 404.6 million individuals worldwide and resulting in nearly 236,786 deaths in 2019 [(Stamm and Norrby, 2001), (Zeng et al., 2022)]. UTIs remain a significant cause of healthcare-associated infections (HAIs), and constitute 23% of infections acquired within the intensive care unit (ICU) until now (Chenoweth, 2021). Actually, UTIs are also the second most common HAIs in China, comprising approximately 11.29% of cases (Wang et al., 2018).The prevalence of UTIs in individuals with T2DM varies depending on the population and diagnostic criteria used. A recent systematic review and meta-analysis reported UTIs prevalence in T2DM was 11.5%, and higher rates were observed among women and those with poorly controlled diabetes. However, certain subgroups, such as older adults and individuals with diabetes-related complications like neuropathy and nephropathy, may have an even higher prevalence of UTIs (Salari et al., 2022). Overall, the rate of UTIs event was 87.3 events per 1000 patient-years among T2DM patients in Germany in a real-world setting (Wilke et al., 2015). A retrospective study showed that the prevalence of UTIs with T2DM was 11.2% in China (He et al., 2018). The increased risk of UTIs in individuals with T2DM can be attributed to various factors, including hyperglycemia, impaired immune function, and structural changes in the urinary tract (Geerlings, 2008). Moreover, in clinical practice, not all patients can obtain definitive gold standard evidence to diagnose UTIs. For example, some patients may experience symptoms, but their urine leukocyte or bacterial counts are within the normal range. Conversely, others may have bacterial counts that exceed the upper limit of the normal range and positive urine nitrite (NIT) results, despite showing no clinical symptoms.

The co-occurrence of UTIs and T2DM is associated with a high incidence rate. Such comorbidity not only severely affects patients’ quality of life but also leads to considerable medical costs. Additionally, recurrent UTIs may erode patients’ confidence in disease management and control. So it’s significantly important to early detect and treat of UTIs in individuals with T2DM in order to prevent further complications.

Current research on UTIs in patients with T2DM includes treatments, clinical characteristics, medical care and analysis of risk factors (Chua et al., 2017; Karadag Arli and Berivan Bakan, 2018; Hur et al., 2019). Previous studies on UTIs risk factors in T2DM patients have mostly been retrospective case-control studies. Some researchers have developed automated systems to assess the risk of catheter-related UTIs, while others have created tools to evaluate and prevent UTIs associated with catheter use (Chua et al., 2017; Karadag Arli and Berivan Bakan, 2018; Hur et al., 2019). However, these tools are limited in their application and there is currently no personalized tool available to predict UTIs risk in T2DM patients from the perspective of clinical diagnosis and treatment. Overall, there is a need for more research on UTIs risk factors in T2DM patients and the development of personalized tools to better meet their clinical needs.

The early identification of high-risk UTIs patients through a simpler approach holds great promise for improving the quality of life of T2DM. Therefore, this study aims to explore the use of machine learning algorithms to develop a personalized predictive model for UTIs in individuals with T2DM, with the goal of improving early identification of high-risk patients and assisting clinical decision-making.

## 2 Materials and methods

### 2.1 Literature review

To comprehensively and systematically collect data for modeling, a literature review was conducted to investigate the factors influencing UTIs in T2DM patients.

#### 2.1.1 Inclusion criteria

(1) Study population are T2DM; (2) Outcome indicators are UTI-related factors; (3) Research categories included case-control studies, cohort studies, cross-sectional studies, and randomized controlled trials (RCTs).

#### 2.1.2 Exclusion criteria

(1) Study population consisted of pregnant women, minors, or patients with tumors; (2) Literature types included conference papers, reviews, systematic reviews, among other secondary research types; (3) Literature contents included animal experiment, pharmacological study and manufacturing processes; (4) Literature that could not be obtained in full text.

#### 2.1.3 Literature search strategy

A comprehensive literature search was conducted on PubMed, Embase, Web of Science, CNKI, WanFang, and SinoMed databases from their inception to 1 July 2022, with no language or geographic restrictions. The search strategy included a combination of subject and free terms, using keywords such as “Type 2 Diabetes Mellitus,” “Urinary Tract Infections,” “Influencing factor” and “Risk factor”.

### 2.2 Data sources and collection

This study included patients who were hospitalized to Sichuan Academy of Medical Sciences & Sichuan Provincial People’s Hospital between September 1, 2018 and August 30, 2021, with a diagnosis of T2DM without UTIs at admission and a diagnosis of UTIs at discharge. The exclusion criteria were as follows: (1) patients with type 1 diabetes mellitus, underage, pregnancy, tumors, or other infections; (2) patients who died during hospitalization; (3) patients with incomplete diagnosis and treatment data. Identifying information such as names, phone numbers, and home addresses will be anonymized to ensure patient confidentiality. And this study has been approved by the Medical Ethics Committee of Medical Sciences & Sichuan Provincial People’s Hospital.

### 2.3 Data pre-processing

#### 2.3.1 Data pre-screening

In this study, the following steps were performed for data pre-screening: (1) Deletion of variables with missing data proportions greater than 90%. (2) Deletion of variables with single category proportions greater than 90%. (3) Deletion of variables with a coefficient of variation less than 0.1.

#### 2.3.2 Data imputation

Four methods were employed for data imputation: (1) Deletion: columns and rows with missing data were removed. (2) Simple imputation: arithmetic mean or median was used to impute continuous variables, mode for categorical variables. (3) Random forest (RF) imputation: the missing values in each column were predicted using a RF model. (4) Improved RF imputation: columns with missing data were sorted in ascending order and imputed by RF model next (Glickenstein et al., 2021).

#### 2.3.3 Data balancing

If the sample is imbalanced, with a difference in the number of positive and negative samples greater than two-fold, balancing is required. (1) Random over-sampling: duplicate the minority class samples to balance. (2) Random under-sampling: Randomly remove samples from the majority class to balance. (3) Synthetic minority oversampling technique (SMOTE): synthesize and supplement new samples from a small amount of original data. (4) borderline SMOTE: an improved algorithm based on SMOTE that only uses minority class samples on the border to synthesize new samples, thus improving the distribution of class samples.

#### 2.3.4 Feature selection

Feature selection is a crucial step in model building after data balancing. It removes redundant and biases variables to produce more accurate and meaningful research conclusions. (1) No selection. (2) Lasso selection: a linear regression-based feature selection method that accurately selects important variables (Tibshirani, 1997). (3) Boruta selection: using RF algorithms to extract feature variables (Motamedi et al., 2022).

### 2.4 Model establishment

Through different data imputation, data balancing and feature selection, 48 data sets were obtained and 18 machine learning algorithms were used on each dataset, respectively. The 18 algorithms including Logistic Regression, Stochastic Gradient Descent (SGD), K-nearest neighbor (KNN), Linear Discriminant Analysis (LDA), decision tree (DT), Gaussian Naïve Bayes, Multinomial Naive Bayes, Bernoulli Naive Bayes, Passive Aggressive, AdaBoost, Quadratic Discriminant Analysis (QDA), Bagging, Support Vector Machine (SVM), RF, Extra Tree, Gradient Boosting, eXtreme gradient boosting (XGBoost), Ensemble Learning (Wu et al., 2022; Xingwei et al., 2022).

The whole process of model establishment was as follows:

(1) The data was divided into a training set and a test set in a ratio of 4:1. The training set was used to build models, and the test set was used to assess model performance.
(2) Ten-fold cross-validation was conducted on the training set to internally validate the model, and evaluated the impact of different data processing methods or machine learning algorithms on model predictive performance by applying 200 Bootstrapping samples from the test set.
(3) The model exhibiting the highest performance was chosen.

### 2.5 Model validation

**FIGURE 1:**
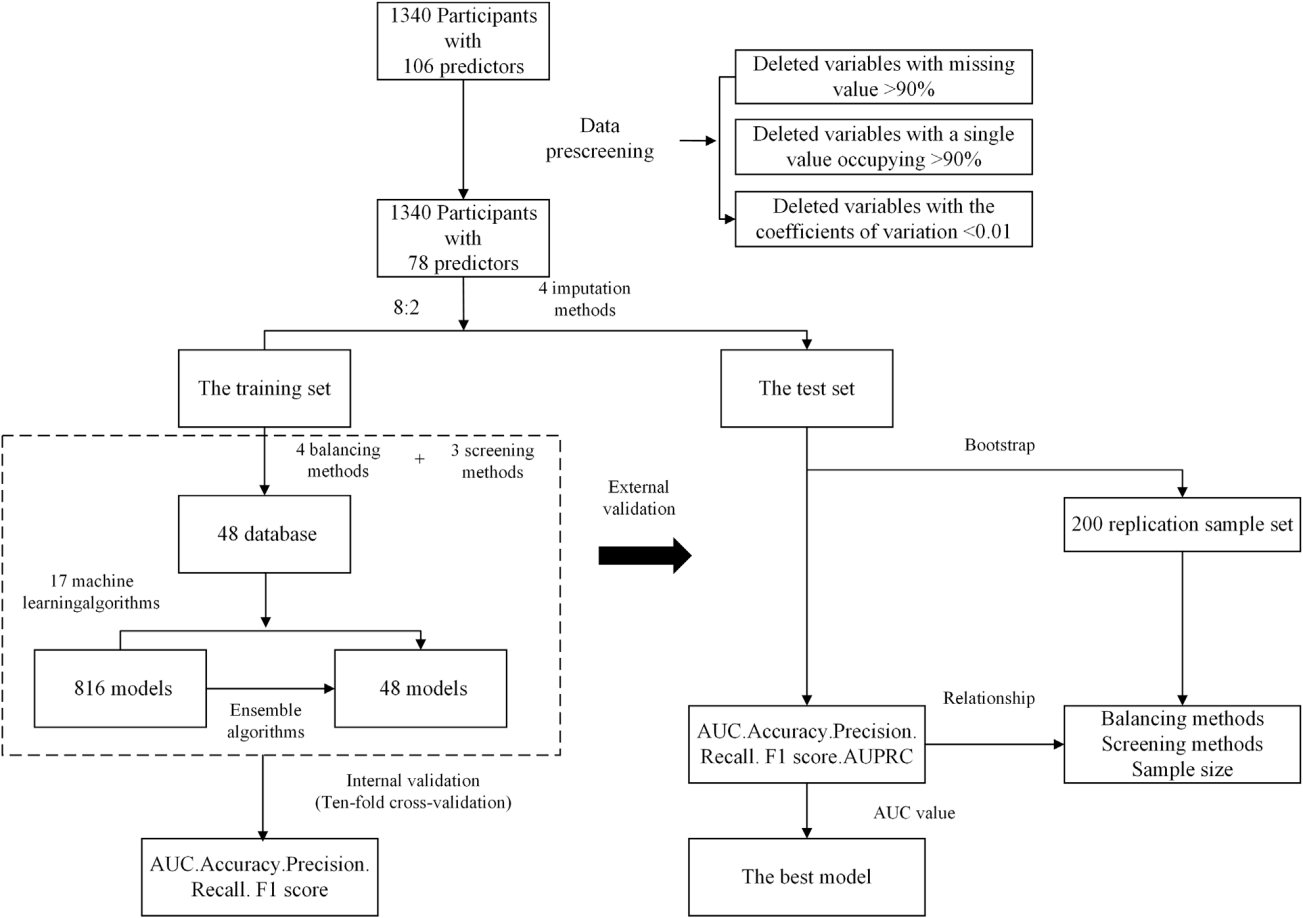
Overview of the modeling process.

AUC (area under the receiver operating characteristic curve), accuracy, precision, recall rate, and F1 value were used to evaluate the model’s predictive performance. SHapley Additive exPlanations (SHAP) was used to explain variable contributions to the model. The modeling process is shown in Figure 1. A total of 864 prediction models were built based on different imputation, balancing and feature selection methods. The top five models with the largest AUC were compared, and the best one was chosen to create a personalized prediction model for UTIs in T2DM.

Insufficient sample size for modeling may lead to bad test efficiency. To evaluate the impact of sample sizes on model performance, subsets of 10%, 20%, 30%, up to 100% were randomly extracted from the training set using Bootstrapping. A model was built for each subset, and this process was repeated 100 times. The AUC value calculated from the testing set was used to evaluate the performance of each model and determine the optimal sample size for the study. Additionally, decision curve analysis (DCA) was used to access the model performance.

### 2.6 Build a web-based prediction platform

Based on the previous steps, we can finally construct a prediction model and build a web-based prediction platform. The information of patients’ individual factors, disease factors, medication factors, laboratory tests and other covariates that are highly correlated with the occurrence of UTIs in T2DM are inputted into the platform, and we finally get the incidence of UTIs in T2DM.

### 2.7 Statistical analysis

Categorical variables were presented as percentages and counts, while continuous variables were expressed as mean ± standard deviation (SD). Univariate analysis was performed using analysis of variance (ANOVA) and rank sum test. The statistical analysis was carried out using the “stats” module in Python 3.8, while model development was performed using the “sklearn” library in Python 3.8.

## 3 Results

### 3.1 Results of literature search

**FIGURE 2:**
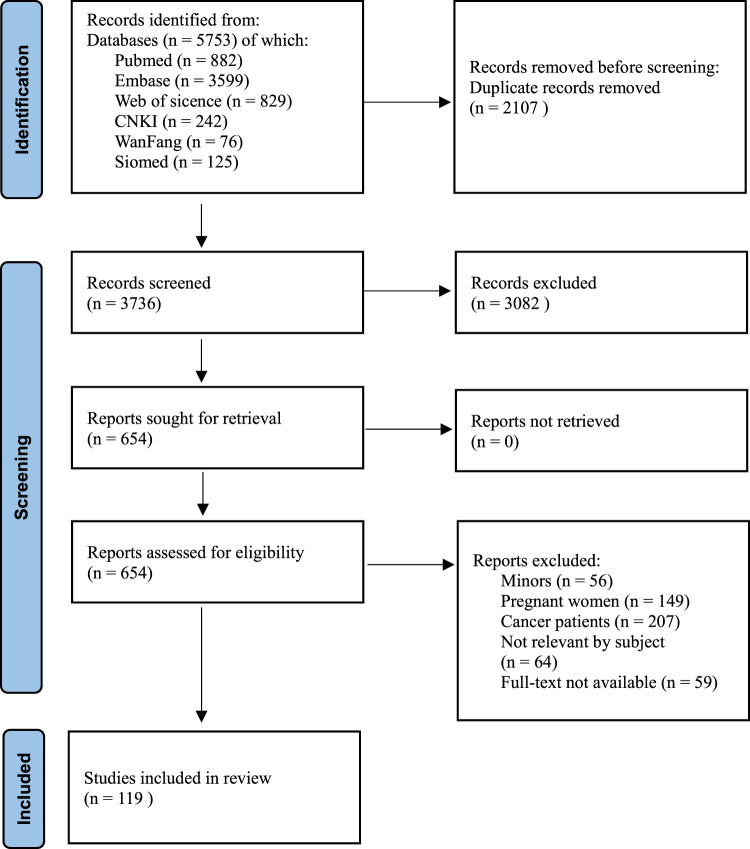
The flowchart of literature selection process.

Based on the search strategy, a total of 5,753 articles were identified and 2,017 duplicates were removed. The titles and abstracts of the remaining 3,736 articles were screened against the inclusion and exclusion criteria, resulting in the exclusion of 3082 articles and leaving 654 articles for full-text screening. After a rigorous review of the full-text, 535 articles were excluded, leaving a final total of 119 articles included in the analysis. The study selection process is presented in a flowchart (see Figure 2). The specific information of the literatures is shown in Supplementary Table S1.

### 3.2 Data collection

**FIGURE 3:**
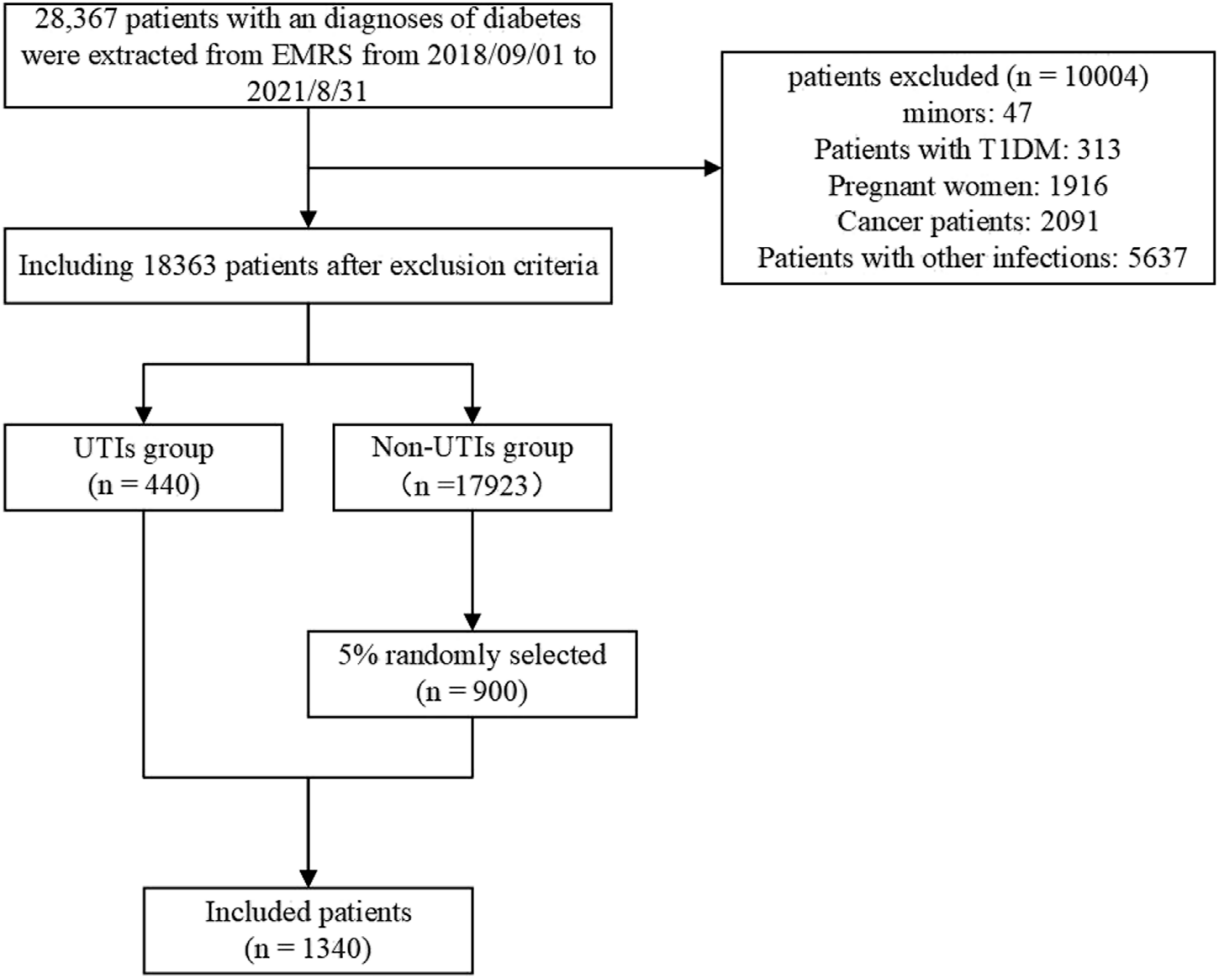
Data screening process flowchart. EMRS, Electronic Medical Record System.

The study extracted a total of 28,367 hospitalized patients with an admission diagnosis of T2DM. After excluding duplicate patients and those with type 1 diabetes mellitus, underage, pregnancy, tumor, or combined with other infections, a total of 18,363 patients were included, of which 440 patients were diagnosed with UTIs at discharge and 17,923 were not. A control group was randomly selected from the non-UTIs group at a rate of 5%, resulting in 900 patients. Ultimately, a total of 1,340 patients were included for model construction. The outline of screening procedures is illustrated in Figure 3. This study finally included 106 variables for analysis and the baseline characteristics is shown in Supplementary Table S2. The principle of variable assignment is shown in Supplementary Table S3.

### 3.3 Data pre-processing

After removing columns that met the deleting criteria, 78 variables were retained and 28 variables were deleted. Then, four data imputation methods were used for replacing missing data. Next, four data balancing methods were used for imbalanced variables. After data pre-screening, data imputation and data balancing, the variables were screened using the no selection, lasso and boruta methods (the details are shown in Supplementary Table S4).

### 3.4 Model establishment

This study developed 864 prediction models using 18 machine learning algorithms and 48 data sets, and evaluated their performance using 10-fold cross-validation. To assess the effect of different data processing methods and machine learning algorithms on model performance, we used 200 Bootstrapping samples from the test set. The results showed that model performance varied depending on data filling, balancing, variable selection, and machine learning algorithm (the details are shown in Supplementary Table S5).

### 3.5 Model evaluation

The model performance was evaluated using AUC, accuracy, precision, recall rate, and F1 value and the area under the precision-recall curve (AUPRC). The top five performing models were selected, and Model 1 demonstrated the best performance with an AUC of 0.9789 and an AUPRC of 0.9585. In the five best models, the data filling method used is no filling, the data balancing method is mainly random over-sampling or random under-sampling, all three feature screening methods are used, and the best machine learning algorithms are gradient boosting.

**FIGURE 4:**
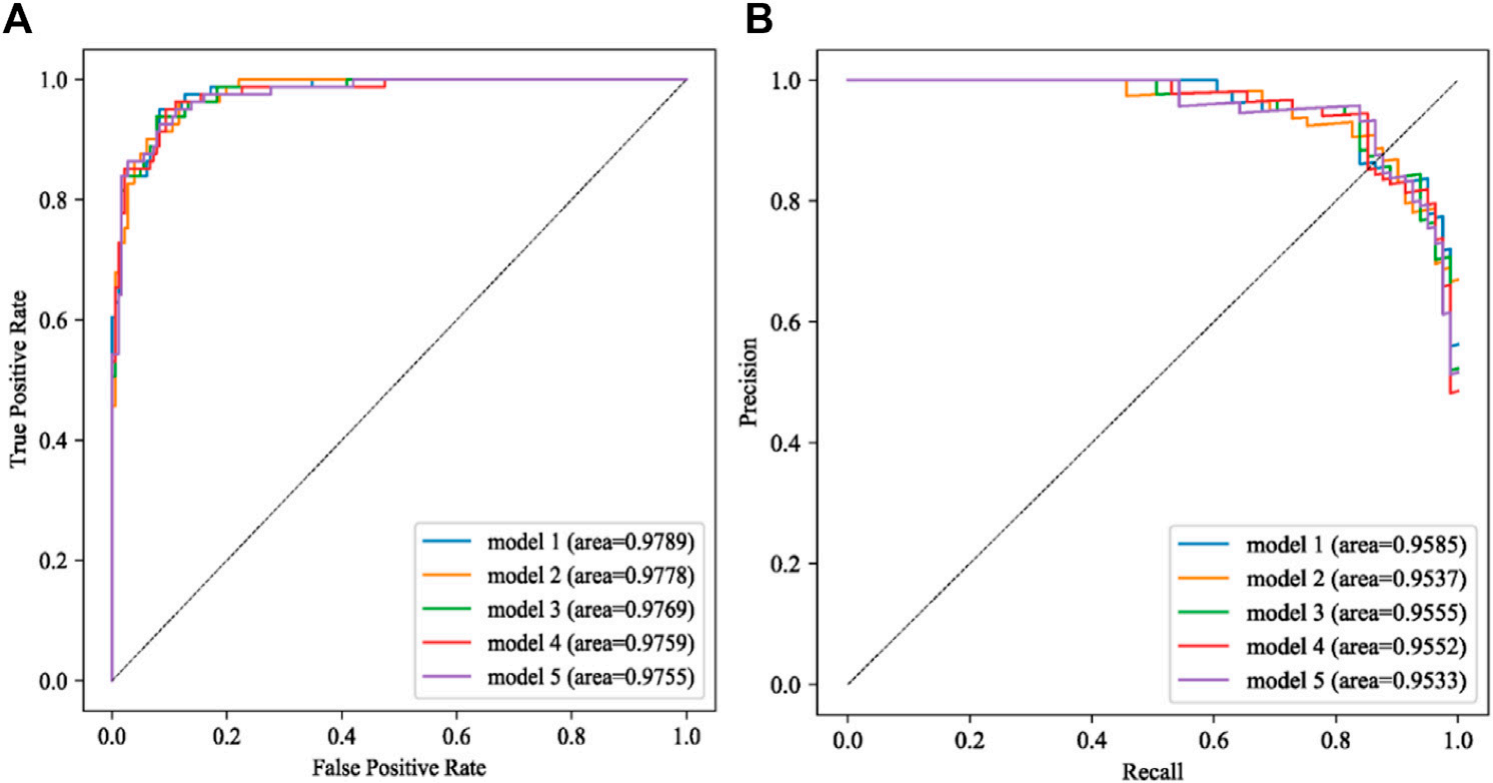
The results of AUC **(A)** and AUPRC **(B)** in the best five models.

**FIGURE 5:**
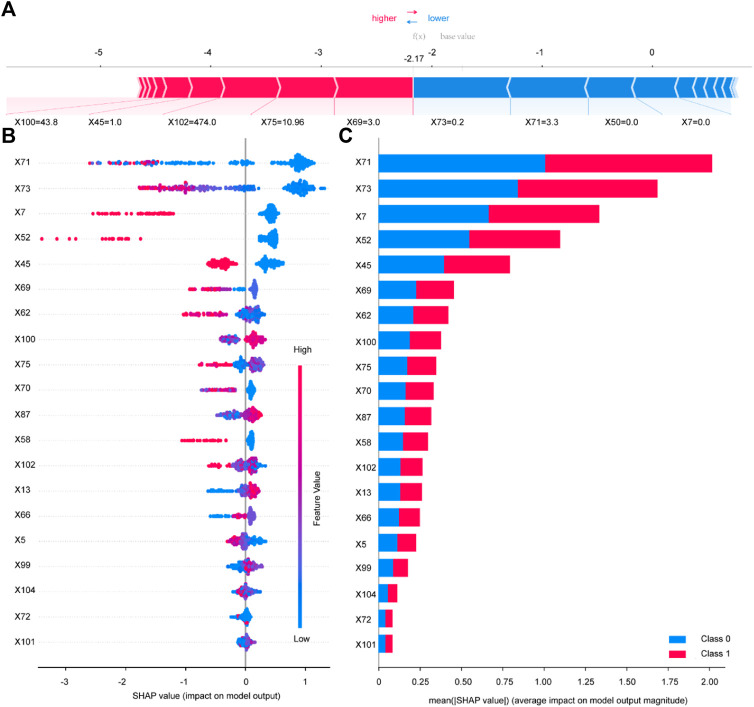
Variable contribution to the model by SHAP Value. Contribution of each feature value in one sample **(A)**. SHAP summary plot of the top 20 variables of the best model **(B)**. Absolute average of SHAP value of the top 20 variables of the best model **(C)**. X5 Length of Stay; X7 History of UTIs; X13 SBP; X45 Insulin; X50 TZD; X52 SGLT-2i; X58 Diuretics; X62 Fasting plasma glucose; X66 Urinary protein; X69 Urine occult blood; X70 Leukocyte esterase; X71 Urine Leukocyte Counts; X72 RBC in Urine; X73 Urine epithelial cells counts; X75 Neutrophil Count; X87 Hb; X99 TBil; X100 eGFR; X101 Urea; X102 UA; X104 U/C.

**FIGURE 6:**
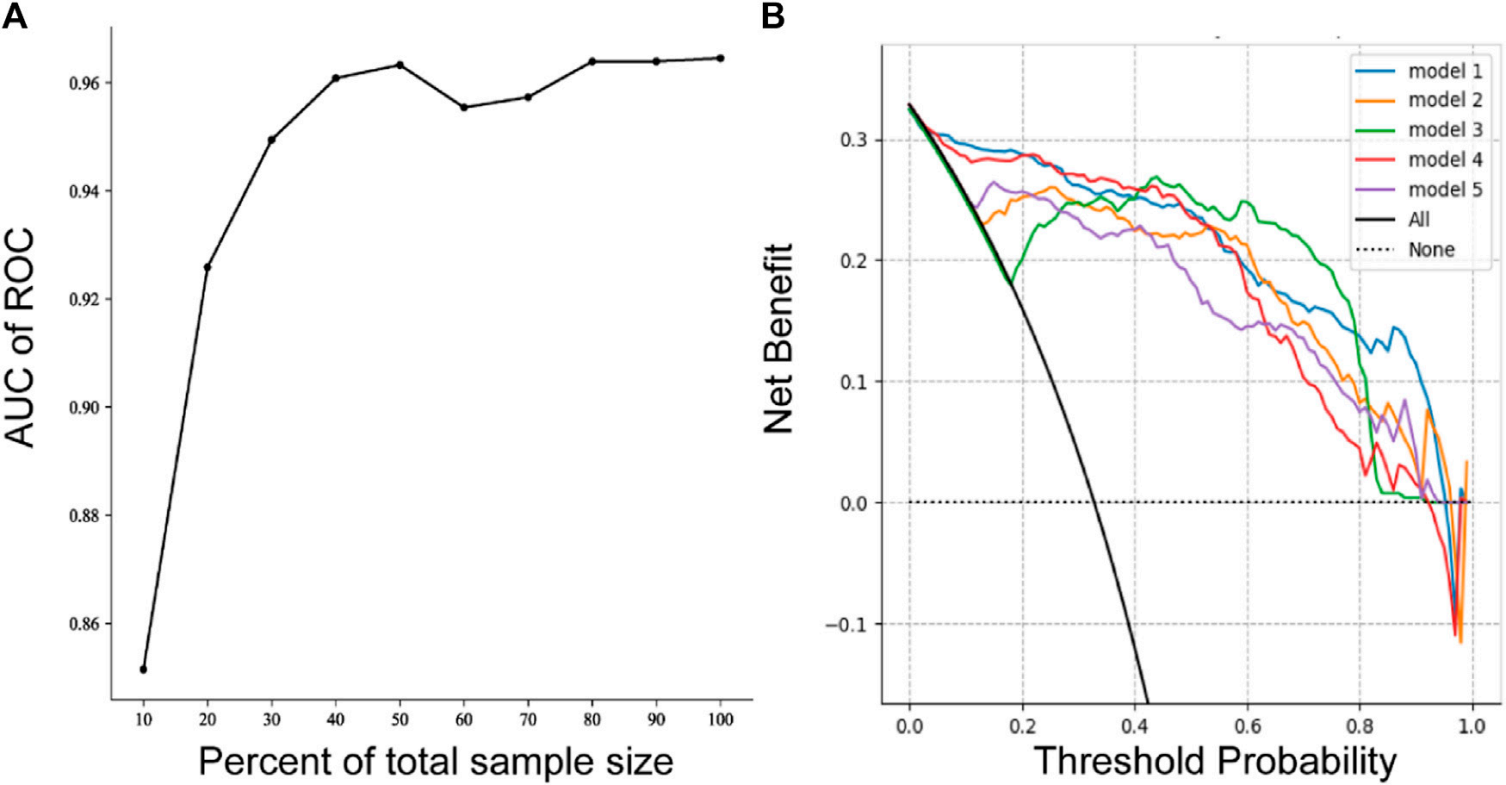
Sample size validation **(A)** and DCA plots of five models **(B)**.

The ROC (receiver operating characteristic curve) for the top five models is presented in Figure 4. The best predictive performance metrics are presented in Table 1. The SHAP value was used to explain the contribution of variables to the model. The importance of each variable to the final prediction model was shown in Figure 5. And the SHAP value of each feature in each sample was calculated and plotted of the top 20 (see Figure 5). This plot explains how high and low variable values were in relation to SHAP values. For the prediction model, the higher the SHAP value of a variable, the more likely UTIs occurs. As the sample size increased, the AUC values of the testing set also increased and the graph showed a gradually flattened trend (Figure 6), indicating that our study had a sufficient sample size. DCA also showed excellent predictive performances (Figure 6).

**TABLE 1:** The predictive performance of top five performing models.

Model ID	AUC	Accuracy	Precision	Recall	F1Score	AUPRC
1	0.9789	0.9237	0.9552	0.7901	0.8649	0.9585
2	0.9778	0.9237	0.8588	0.9012	0.8795	0.9537
3	0.9769	0.916	0.8734	0.8519	0.8625	0.9555
4	0.9759	0.9313	0.92	0.8519	0.8846	0.9552
5	0.9755	0.9275	0.9559	0.8025	0.8725	0.9533

### 3.6 A pattern tool for prediction model

**FIGURE 7:**
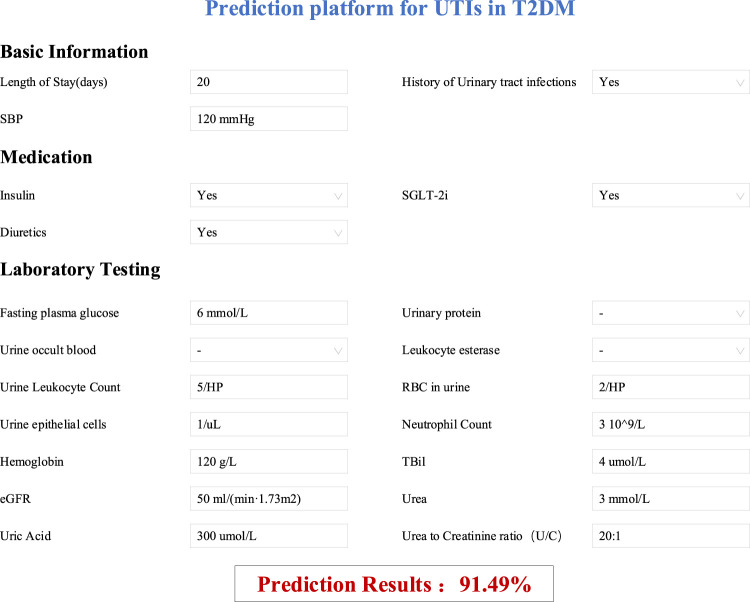
Prediction platform for UTIs in T2DM.

According to the best model, a prediction platform for the UTIs of T2DM patients has been developed, the function of the prediction model was shown in Figure 7. For example, a patient, who has been hospitalized for 20 days and has a history of UTIs, with a systolic blood pressure (SBP) of 120 mmHg, was receiving combined treatment with insulin, SGLT2 inhibitors, and diuretics. Considering the patient’s other laboratory test results related to UTIs, the likelihood of UTIs was estimated to be 91.49%. This prediction platform, combined with the professional judgment of clinicians on the outcome, can assist doctors to make better clinical decisions.

## 4 Discussion

In our study, a total of 1340 patients with T2DM were included to build models. Four data imputing methods, four data balancing methods and three feature screening methods were used to build 48 datasets, and 18 machine learning algorithms were used to develop 864 machine learning models. AUC, accuracy, precision, recall, F1 score, and AUPRC were used to evaluate the performance of the models. The results showed that our model performed better than models built using conventional statistical methods, such as univariate analysis and multivariate binary logistic regression. For example, Maria *et al* established a UTIs prediction model with T2DM whose AUC was 0.862 (35), which is also one of the few UTIs prediction models with T2DM currently.

UTIs is a common infection in patients with T2DM. Early prediction of UTIs occurrence can minimize its occurrence. Multiple machine learning algorithms and feature selection methods were employed to construct a UTIs prediction model. The model can aid in early intervention measures for high-risk individuals by adjusting the use of hypoglycemic agents and controlling blood glucose levels to reduce the incidence of UTIs.

According to the results, the important features of UTIs in T2DM mainly include the following aspects: UTIs-related inflammatory indicators (including leucocyte, urinary epithelial cells, urinary leukocyte, etc.), medication use (mainly SGLT2 inhibitors, insulin, etc.), severity of comorbidities (history of UTIs, diabetes and hypertension), blood routine indicators (neutrophil count), and other indicators (length of hospital stay and eGRF).

Apparently, infection markers in urine analysis are directly associated with UTIs. Although some patients may present with asymptomatic bacteriuria (ASB), most UTIs in T2DM patients exhibit elevated levels of infection markers in urine analysis (Sharma et al., 2017). Additionally, this study demonstrated that blood routine examination, such as neutrophil count, may also serve as potential indicators, which is consistent with the findings of Fatemeh et al. (Saheb Sharif-Askari et al., 2020).

Many RCTs and clinical reviews indicated that the use of SGLT-2 inhibitors was associated with an increased risk of UTIs (Clar et al., 2012; Musso et al., 2012; Berhan and Barker, 2013; Vasilakou et al., 2013; Liu et al., 2015b; Dave et al., 2019). The possible mechanism is that SGLT-2 inhibitors can increase the excretion of glucose in urine, providing a better environment for the growth of microorganisms such as fungi and bacteria in the genitourinary tract, leading to an increased risk of UTIs (Geerlings et al., 2014). Furthermore, the severity of T2DM itself exacerbates the risk of UTIs. Therefore, indicators of the control status of T2DM are of significant importance in predicting the occurrence of UTIs. These indicators include fasting blood glucose (FBG) levels, insulin use, and the presence of diabetes-related complications, such as ocular problems caused by diabetes (Wilke et al., 2015). Moreover, the elevation of blood pressure is also identified as a contributing risk factor (Carrondo and Moita, 2020). In addition, the history of UTIs is also very important, because it suggested that the patient may possess susceptibility to recurrent UTIs, which provides crucial insights into the patient’s medical history, immune status, anatomical abnormalities, or pathological changes. And these factors may elevate the risk of future UTIs. Previous studies have demonstrated that a history of UTIs is a strong risk factor for UTIs (Geerlings et al., 2014; Wilke et al., 2015).

Other indicators, including the length of hospital stay and eGFR are also considered risk factors, which is also consistent with the results of some previous studies (Janifer et al., 2009; Wilke et al., 2015; Carrondo and Moita, 2020). eGFR is a possible influencing factor probably because of poorer kidney function status, because the patients were mostly elderly (>64 years) (see Supplementary Table S2). And women are more susceptible to UTIs compared to men, primarily because the female urethra is shorter, which makes it easier for bacteria to invade, however, the importance of gender was not shown in our study, probably because the gender difference was not obvious, and the baseline data showed that the male to female ratio was 1:1 (Gyftopoulos et al., 2019; Czajkowski et al., 2021).

Some study found that invasive procedures increased the risk of UTIs, as they can damage the urethral mucosa and facilitate bacterial entry (Mirone and Franco, 2014; Walker et al., 2017). However, in this study, the proportion of invasive procedures was higher in the non-UTIs group. This discrepancy may be due to the fact that all invasive procedures were included in this study, while other studies only considered invasive procedures related to the genitourinary tract.

## 5 Limitations

This study had several limitations. First, this was a retrospective study, so some variables that could be important for UTIs, such as specific invasive procedures and dietary habits, may not have been obtainable and could affect the predictive performance of the model. Second, although the results of the sample size validation are acceptable, the final sample size included in the study is relatively small. Third, all the data were from the same hospital, so whether the predictive model developed in this study is applicable to other hospitals or populations in other countries, further research is needed. Therefore, further research is needed to determine the applicability of the predictive model developed to other populations.

## 6 Conclusion

We have developed a predictive model for UTIs in T2DM patients based on machine learning. In this process, we utilized various combinations of imputing methods, sampling methods, feature screening methods, and algorithms. Through the establishment of the predictive model, we aim to provide some assistance for the clinical diagnosis and treatment of UTIs in T2DM.

## Data Availability

The original contributions presented in the study are included in the article/Supplementary Material, further inquiries can be directed to the corresponding authors.
