# Supplementary material for: A personalized prediction model for urinary tract infections in type 2 diabetes mellitus using machine learning
Source: Front Pharmacol. 2024 Jan 5;14:1259596. doi: 10.3389/fphar.2023.1259596 (PMC10806526; doi:10.3389/fphar.2023.1259596)
Supplement: Supplementary file 1 [file Table1.DOCX]

Supplementary materials

Table S1 The specific information of the literature(N=119)

| **Year** | **Author** | **Country** | **Influencing factors** | **Sample Size** | |
| --- | --- | --- | --- | --- | --- |
| 2022 | Zhang Y | China | Age、Gender、Alcohol Drinking | 185 | |
| 2022 | Yen FS | China | History of urinary tract infection、History of invasive procedures | 68 | |
| 2022 | Yen wmiil | US | Diabetic Nephropathies、SGLT-2i | 1236 | |
| 2022 | Guan Qingxia | China | Diabetic Retinopathy、Indwelling Catheter、HbA1c | 40 | |
| 2022 | Yen yhnk | Netherlands | Kidney Calculi、SGLT-2i | 84 | |
| 2022 | Waseem S | India | Urinary Calculi、Diabetes duration、Menopause | 245 | |
| 2022 | Tamborlane WV | Denmark | Age、eGFR | 321 | |
| 2022 | Alkabbani W | US | Diabetic Nephropathies、Hospitalization history | 102 | |
| 2021 | Tumminia A | Sweden | History of urinary tract infection、Occupations | 725 | |
| 2021 | Tobe JM | Japan | Urinary Calculi、eGFR、ACEI、Hypertension | 202 | |
| 2021 | Tobe K | France | History of invasive procedures、Diabetic Retinopathy | 382 | |
| 2021 | Kumar A | US | SGLT-2i、TBil | 205 | |
| 2021 | Ko NK | Russia | History of invasive procedures | 1752 | |
| 2021 | Ko S | Poland | SGLT-2i、Osteoporosis | 1025 | |
| 2021 | Kande S | US | Kidney Calculi、TC | 205 | |
| 2021 | Hopf M | Russia | History of urinary tract infection、Dementia | 48 | |
| 2021 | Bhatt DL | Sweden | Diuretics | 96 | |
| 2021 | Aamir AH | Netherlands | Urinary Calculi、Angina Pectoris | 58 | |
| 2020 | Zaidi SMJ | Korea | β-receptor blocker ，Income | 1025 | |
| 2020 | Szlachetka WA | India | SGLT-2i、Creatinine | 425 | |
| 2020 | Liu Xin | China | Age、BMI、Kidney Calculi、invasive procedures | 98 | |
| 2020 | Sriphrapradang | Russia | Urinary albumin、Amputation | 520 | |
| 2020 | Sone H， | Saudi Arabia | HbA1c | 613 | |
| 2020 | Radholm K | Singapore | History of invasive procedures、SGLT-2i | 324 | |
| 2020 | Pratley R | US | Urinary albumin、Metformin | 251 | |
| 2020 | Carrondo MC | Canada | SGLT-2i、Liver Diseases | 52 | |
| 2019 | Yabe D | Japan | SGLT-2i、Insulin | 1025 | |
| 2019 | Wilding J | Switzerland | eGFR、DPP-4 inhibitors | 125 | |
| 2019 | Waragai Y | Russia | Diuretics、Urinary albumin | 235 | |
| 2019 | Wang X | China | History of urinary tract infection、Gout | 258 | |
| 2019 | Terauchi Y | Germany | Diuretics、TBil | 352 | |
| 2019 | Kim EJ | Russia | Urinary Calculi、Urinary albumin | 254 | |
| 2019 | Gill HK | US | Diuretics | 258 | |
| 2019 | Dave CV | Poland | History of invasive procedures、Anemia、Statins | 452 | |
| 2019 | NZM KL | Germany | Diabetic Nephropathies、Insulin | 82 | |
| 2019 | Allegretti AS | Korea | HbA1c | 88 | |
| 2018 | Ahmadi F | Mexico | Urinary albumin | 73 | |
| 2018 | Yasui A | Japan | Diuretics | 68 | |
| 2018 | Yang M | China | Urinary albumin | 42 | |
| 2018 | Hu Xiaoquan | China | HbA1c、Length of Stay、Indwelling Catheter | 1520 | |
| 2018 | Yang M | China | Urinary albumin | 1202 | |
| 2018 | Yang AY | China | History of urinary tract infection | 632 | |
| 2018 | Ueda P | UK | eGFR | 1250 | |
| 2018 | Zheng Yan | China | Age、Diabetes duration、Diabetic Nephropathies、History of invasive procedures、HbA1c | 278 | |
| 2018 | Ueda P | Netherlands | SGLT-2i | 452 | |
| 2018 | Thong KY | Spain | HbA1c | 338 | |
| 2018 | Shestakova MV | Canada | Urinary Calculi、Urinary albumin | 432 | |
| 2018 | Schnee | US | Diuretics | 283 | |
| 2018 | Fioretto P | Sweden | SGLT-2i | 93 | |
| 2018 | Dagogo-Jack S | Italy | History of urinary tract infection | 42 | |
| 2017 | Thewjitcharoen | Japan | Diabetic Nephropathies、SGLT-2i | 1035 | |
| 2017 | Terra S | Korea | Age、Marital Status、Height、Alcohol Drinking | 746 | |
| 2017 | Shiba T | Iran | SGLT-2i | 73 | |
| 2017 | Qin Zhiqian | China | Gender、Age、Diabetes duration、HbA1c | 683 | |
| 2017 | Nichols GA | India | Diuretics、Urinary albumin | 325 | |
| 2017 | Gupta S | Germany | SGLT-2i | 152 | |
| 2017 | Charbonnel B | US | Urinary Calculi、Exercise | 135 | |
| 2017 | Borowczyk M | Netherlands | History of urinary tract infection、eGFR | 982 | |
| 2016 | Yoon KH | Germany | HbA1c | 45 | |
| 2016 | Yang WY | China | History of urinary tract infection、Diuretics | 473 | |
| 2016 | Thomsen RW | Norway | Diuretics | 1326 | |
| 2016 | Sinclair AJ | Japan | History of invasive procedures | 866 | |
| 2016 | Shatskov A | Portugal | Urinary albumin | 1332 | |
| 2016 | Salsali A | US | History of urinary tract infection、Urinary albumin | 205 | |
| 2016 | Romera I | Russia | SGLT-2i、Sexual Behavior | 123 | |
| 2016 | Mathieu C | Sweden | Diabetic Nephropathies、eGFR | 85 | |
| 2016 | Li M | China | SGLT-2i | 450 | |
| 2016 | Kohler S | Spain | History of urinary tract infection | 132 | |
| 2016 | Ishihara H | Netherlands | Kidney Calculi、Rheumatoid arthritis | 410 | |
| 2016 | Zhao X | China | SGLT-2i | 82 | |
| 2016 | Ji L | China | Age、Diuretics | 63 | |
| 2015 | Bode B | Denmark | SGLT-2i | 66 | |
| 2015 | Bailey CJ | Japan | History of urinary tract infection、SGLT-2i | 125 | |
| 2015 | Yamout H | US | HbA1c | 742 | |
| 2014 | Yale JF | Thailand | SGLT-2i | 45 | |
| 2014 | Sykes A | Germany | HbA1c | 123 | |
| 2014 | Stenlöf K | France | SGLT-2i | 163 | |
| 2014 | Sasaki T | India | Urinary albumin、Obesity | 221 | |
| 2014 | Ross S | Sweden | History of invasive procedures | 108 | |
| 2014 | Prasanna Kumar KM | Netherlands | HbA1c | 63 | |
| 2014 | Lehrke M | Kuwait | Diabetic Nephropathies | 108 | |
| 2014 | Ji L | China | History of urinary tract infection | 55 | |
| 2014 | Haring HU | Germany | BMI、Depression | 630 | |
| 2014 | Chuang LM | China | Age、SGLT-2i | 452 | |
| 2013 | Wilding JPH | Australia | History of urinary tract infection | 125 | |
| 2013 | Wilding JP | Mexico | Diuretics、Urinary albumin | 205 | |
| 2013 | Stenlof K | US | Kidney Calculi、Urinary albumin | 68 | |
| 2013 | Sinclair A | Japan | BMI、AST | 82 | |
| 2013 | Schernthaner G | Russia | History of urinary tract infection、Diuretics | 136 | |
| 2013 | Rosenstock J | US | Waist Circumference | 93 | |
| 2013 | Matthaei S | Japan | SGLT-2i | 832 | |
| 2013 | Lavalle-González FJ | France | eGFR | 152 | |
| 2013 | Langkilde A | US | History of invasive procedures | 163 | |
| 2013 | Kaku K | Spain | Diabetic Nephropathies、Diuretics | 223 | |
| 2013 | Johnsson KM | Poland | History of urinary tract infection、Urinary albumin | 270 | |
| 2013 | Januszewicz A | Australia | Diuretics | 149 | |
| 2013 | Al-Rubeaan KA | Sweden | SGLT-2i | 382 | |
| 2012 | Wilding JPH | Japan | eGFR | 62 | |
| 2012 | Chen Xiaoyan | China | 2-hour postprandial plasma glucose、Chronic complications | 60 | |
| 2012 | Li Yan | China | Age、Gender、disease duration、HbA1c、Indwelling Catheter | | 108 |
| 2012 | Feng Yongjun | Germany | Antibiotics、Gender、HbA1c | 350 | |
| 2012 | Wilding J | France | Diabetic Nephropathies | 78 | |
| 2012 | Wlm J | Denmark | History of invasive procedures | 69 | |
| 2012 | Schernthaner G | UK | SGLT-2i | 91 | |
| 2012 | Kaku K | US | History of urinary tract infection、Interleukin-6 | 305 | |
| 2012 | Hirji I | Italy | Diabetic Nephropathies、BUN | 108 | |
| 2011 | Riggs MM | UK | SGLT-2i、C-Peptide | 98 | |
| 2010 | Wilding J | US | Diuretics、Dyslipidemias | 362 | |
| 2010 | Strojek K | Thailand | History of invasive procedures | 102 | |
| 2010 | Schlichting N | India | Diabetic Nephropathies、Urinary albumin | 48 | |
| 2010 | Sanden AK | US | Diuretics、Hyperuricacidemia | 94 | |
| 2010 | Oberbach A | Norway | History of urinary tract infection、Blood pressure | 312 | |
| 2010 | Gorter KJ | Sweden | SGLT-2i、Obstructive nephropathy | 652 | |
| 2009 | Venmans LMAJ | Pakistan | Age、TC、TG | 224 | |
| 2009 | Venmans L | Canada | Diuretics、HDL/LDL | 63 | |
| 2009 | Lamloum SM | US | History of urinary tract infection、Kidney Calculi | 207 | |
| 2008 | Schnporger | Japan | Diuretics、Urinary Incontinence | 301 | |
| 2007 | Venmans LMAJ | Melbourne | History of invasive procedures、Urogenital Diseases | 53 | |
| 2005 | Ribera-Montes Mdel C | UK | SGLT-2i、Gastrointestinal Diseases | 148 | |

Table S2 The baseline characteristics of 2 groups

| **Variables** | | **UTIs(N=440)** | **Non-UTIs(N=900)** |
| --- | --- | --- | --- |
|  | | **Mean±SD or Frequency (%)** | |
| Gender |  |  |  |
|  | Male | 199（45.20） | 506(56.20) |
|  | Female | 241(54.80) | 394(43.80) |
| Age |  | 64.2±13.89 | 64.6±12.48 |
| Menopausal or not |  |  |  |
|  | Yes | 222（92.11） | 368(40.90) |
|  | No | 19(4.30) | 26(2.90) |
|  | Male | 199（45.20） | 506(56.20) |
| Marital status |  |  |  |
|  | Married | 411(93.40) | 862(95.80) |
|  | Unmarried | 29(6.60) | 38(4.20) |
| Length of stay (days) |  | 11.1±5.84 | 8.2±7.24 |
| Diabetes duration(years) |  | 9.6±7.86 | 7.2±6.94 |
| History of UTIs |  |  |  |
|  | Yes | 215(48.90) | 33(3.70) |
|  | No | 225(51.50) | 867(96.30) |
| History of invasive procedures |  |  |  |
|  | Yes | 140(31.80) | 543(60.30) |
|  | No | 300(68.20) | 357(39.70) |
| BMI |  | 24.3±3.54 | 24.2±2.97 |
| Smoking history |  |  |  |
|  | Yes | 102(23.18) | 202(22.40) |
|  | No | 335(76.13) | 698(77.60) |
|  | Unknown | 3(0.68) | 0(0.00) |
| Drinking history |  |  |  |
|  | Yes | 80(18.18) | 131(14.60) |
|  | No | 357(81.14) | 769(85.40) |
|  | Unknown | 3(0.68) | 0(0.00) |
| DBP (mmHg) |  | 81.0±20.35 | 77.2±11.97 |
| SBP (mmHg) |  | 133.2±25.40 | 139.4±19.70 |
| Hypertension |  |  |  |
|  | Yes | 265(60.20) | 491(54.60) |
|  | No | 175(39.80) | 409(45.40) |
| Arrhythmias |  |  |  |
|  | Yes | 8(1.80) | 50(5.60) |
|  | No | 432(98.20) | 850(94.40) |
| Heart failure |  |  |  |
|  | Yes | 10(2.30) | 18(2.00) |
|  | No | 430(97.70) | 882(98.00) |
| Diabetic peripheral vascular diseases |  |  |  |
|  | Yes | 139(31.60) | 139(15.40) |
|  | No | 301(68.40) | 761(84.60) |
| Coronary artery disease |  |  |  |
|  | Yes | 93(21.10) | 254（28.20） |
|  | No | 347(78.90) | 646(71.80) |
| Other cardiovascular diseases |  |  |  |
|  | Yes | 75(17.00) | 95(10.60) |
|  | No | 365(83.00) | 805(89.4) |
| Diabetic nephropathies |  |  |  |
|  | Yes | 121(27.50) | 62(6.90) |
|  | No | 319(72.50) | 838(93.10) |
| Nephrosis |  |  |  |
|  | Yes | 74(16.80) | 123(13.70) |
|  | No | 366(83.20) | 777(86.30) |
| Kidney calculi |  |  |  |
|  | Yes | 67(15.20) | 34(3.80) |
|  | No | 373(84.80) | 866(96.20) |
| Urinary calculi |  |  |  |
|  | Yes | 82(18.60) | 22(2.40) |
|  | No | 358(81.40) | 878(97.60) |
| Urinary incontinence |  |  |  |
|  | Yes | 3(0.70) | 0(0.00) |
|  | No | 437(99.30) | 900(100.00) |
| Other urologic diseases |  |  |  |
|  | Yes | 141(32.00) | 103(11.00) |
|  | No | 299(68.00) | 797(89.00) |
| Diabetic retinopathy |  |  |  |
|  | Yes | 69(15.70) | 138(15.30) |
|  | No | 371(84.30) | 762(84.70) |
| Other eye diseases |  |  |  |
|  | Yes | 31(7.00) | 248(28.00) |
|  | No | 409(93.00) | 652(72.00) |
| Diabetic peripheral neuropathies |  |  |  |
|  | Yes | 145(33.00) | 147(16.30) |
|  | No | 295(67.00) | 753(83.70) |
| Other nervous system diseases |  |  |  |
|  | Yes | 22(5.00) | 78(8.70) |
|  | No | 418(95.00) | 822(91.30) |
| Liver diseases |  |  |  |
|  | Yes | 105(23.90) | 153(17.00) |
|  | No | 335(76.10) | 747(83.00) |
| Gastrointestinal diseases |  |  |  |
|  | Yes | 18(4.10) | 6(0.70) |
|  | No | 422(95.90) | 894(99.30) |
| Other digestive system diseases |  |  |  |
|  | Yes | 40(9.10) | 104(11.60) |
|  | No | 400(90.90) | 796(88.40) |
| Other urogenital diseases |  |  |  |
|  | Yes | 42(9.50) | 32(3.60) |
|  | No | 398(90.50) | 868(96.40) |
| Dyslipidemias |  |  |  |
|  | Yes | 77(17.50) | 156(17.30) |
|  | No | 363(82.50) | 744(82.70) |
| Osteoporosis |  |  |  |
|  | Yes | 28(6.40) | 31(3.40) |
|  | No | 412(93.60) | 869(96.60) |
| Hyperuricacidemia |  |  |  |
|  | Yes | 25(5.70) | 14(1.60) |
|  | No | 415(94.30) | 886(98.40) |
| Other endocrine system diseases |  |  |  |
|  | Yes | 90(20.50) | 73(8.10) |
|  | No | 350(79.50) | 827(91.90) |
| Dementia |  |  |  |
|  | Yes | 7(1.60) | 4(0.40) |
|  | No | 433(98.40) | 896(99.60) |
| Depression |  |  |  |
|  | Yes | 1(0.20) | 18(2.00) |
|  | No | 439(99.80) | 882(98.00) |
| Other mental disorders |  |  |  |
|  | Yes | 1(0.20) | 8(0.90) |
|  | No | 439(99.80) | 892(99.10) |
| Rheumatoid arthritis |  |  |  |
|  | Yes | 2(0.50) | 5(0.60) |
|  | No | 438(99.50) | 895(99.40) |
| Other immune system diseases |  |  |  |
|  | Yes | 7(1.60) | 4(0.40) |
|  | No | 433(98.40) | 896(99.60) |
| Anemia |  |  |  |
|  | Yes | 50(11.40) | 41(4.60) |
|  | No | 390(88.60) | 859(95.40) |
| Other hematologic diseases |  |  |  |
|  | Yes | 4(0.90) | 14(1.60) |
|  | No | 436(99.10) | 886(98.40) |
| Insulin |  |  |  |
|  | Yes | 295(67.00) | 361(40.10) |
|  | No | 145(33.00) | 539(59.90) |
| Metformin |  |  |  |
|  | Yes | 117(26.60) | 212(23.60) |
|  | No | 323(73.40) | 688(76.40) |
| Sulfonylurea |  |  |  |
|  | Yes | 51(11.60) | 72(8.00) |
|  | No | 389(88.40) | 828(92.00) |
| Glinides |  |  |  |
|  | Yes | 23(5.20) | 43(4.80) |
|  | No | 417(94.80) | 857(95.20) |
| α-glucosidase inhibitor |  |  |  |
|  | Yes | 69(15.70) | 140(15.60) |
|  | No | 371(84.30) | 760(84.40) |
| TZD |  |  |  |
|  | Yes | 18(4.10) | 18(2.00) |
|  | No | 422(95.90) | 882(98.0) |
| DPP-4 inhibitors |  |  |  |
|  | Yes | 100(22.70) | 72(8.00) |
|  | No | 340(77.30) | 828(92.0) |
| SGLT-2i |  |  |  |
|  | Yes | 152(34.50) | 2(0.20) |
|  | No | 288(65.50) | 898(99.80) |
| GLP-1 receptor agonist |  |  |  |
|  | Yes | 72(16.40) | 7(0.80) |
|  | No | 368(83.60) | 893(99.20) |
| Finasteride |  |  |  |
|  | Yes | 76(17.30) | 20(2.20) |
|  | No | 364(82.70) | 880(97.80) |
| Calcium channel blocker |  |  |  |
|  | Yes | 110(25.00) | 188(20.90) |
|  | No | 330(75.00) | 712(79.10) |
| ACEI |  |  |  |
|  | Yes | 12(2.70) | 12(1.30) |
|  | No | 428(97.30) | 888(98.70) |
| ARB |  |  |  |
|  | Yes | 66(15.00) | 132(14.70) |
|  | No | 374(85.00) | 768(85.30) |
| Diuretics |  |  |  |
|  | Yes | 109(24.80) | 36(4.00) |
|  | No | 331(75.20) | 864(96.00) |
| β-blocker |  |  |  |
|  | Yes | 28(6.40) | 82(9.10) |
|  | No | 412(93.60) | 818(90.90) |
| Statins |  |  |  |
|  | Yes | 124(28.20) | 288(32.00) |
|  | No | 316(71.80) | 612(68.00) |
| NSAIDs |  |  |  |
|  | Yes | 57(13.00) | 215(23.90) |
|  | No | 383(87.00) | 685(76.10) |
| Fasting plasma glucose |  | 11.2±6.24 | 8.0±3.48 |
| HbA1c |  | 9.0±2.66 | 8.1±2.28 |
| Microalbuminuria |  | 910.1±3252.95 | 363.1±1131.82 |
| 24-hour urinary protein quantification |  | 3.6±6.64 | 2.4±3.27 |
| Urinary protein |  |  |  |
|  | - | 77(17.50) | 76（8.40） |
|  | ± | 194(44.10) | 742（82.40） |
|  | 1+ | 84(19.10) | 43(4.80) |
|  | 2+ | 48(10.90) | 29(3.20) |
|  | 3+ | 26(5.90) | 8(0.90) |
|  | 4+ | 11(2.50) | 2(0.30) |
| Urine glucose |  |  |  |
|  | - | 22(5.00) | 49(5.40) |
|  | ± | 220(50.00) | 581(64.60) |
|  | 1+ | 30(6.80) | 53(5.90) |
|  | 2+ | 36(8.20) | 57(6.30) |
|  | 3+ | 45(10.20) | 79(8.80) |
|  | 4+ | 87(19.80) | 81(9.00) |
| Urine ketones |  |  |  |
|  | - | 3(0.70) | 10(1.10) |
|  | ± | 412(93.60) | 854(94.90) |
|  | 1+ | 16(3.60) | 24(2.70) |
|  | 2+ | 7(1.60) | 12(1.30) |
|  | 3+ | 2(0.50) | 0(0.00) |
| Urine occult blood |  |  |  |
|  | - | 68(15.50) | 42(4.70) |
|  | ± | 195(44.30) | 804(89.30) |
|  | 1+ | 69(15.70) | 33(3.70) |
|  | 2+ | 53(12.00) | 9(1.00) |
|  | 3+ | 55(12.50) | 12(1.30) |
| Urine Leukocyte |  |  |  |
|  | - | 22(5.00) | 24(2.70) |
|  | ± | 160(36.40) | 791(87.90) |
|  | 1+ | 62(14.10) | 41(4.60) |
|  | 2+ | 83(18.90) | 27(3.00) |
|  | 3+ | 113(25.60) | 17(1.80) |
| Urine Leukocyte Counts(cells/uL) |  | 619.1±3270.77 | 17.3±86.16 |
| RBC in Urine(cells/uL) |  | 391.0±2445.96 | 33.4±395.33 |
| Urine epithelial cells Count(cells/uL) |  | 5.4±3.85 | 2.8±4.16 |
| Leukocyte Count(WBC)（10^9/L） |  | 7.6±3.25 | 6.6±2.09 |
| Neutrophil Count（10^9/L） |  | 5.5±3.23 | 4.4±1.82 |
| Neutrophils（％） |  | 68.0±11.95 | 65.2±9.98 |
| Lymphocyte Count（10^9/L） |  | 1.6±0.66 | 1.6±0.61 |
| Lymphocytes（％） |  | 22.7±10.25 | 25.4±8.72 |
| Monocyte Count（10^9/L） |  | 0.5±0.32 | 0.4±0.17 |
| Monocytes（％） |  | 6.8±3.02 | 6.7±2.33 |
| Eosinophil Count（10^9/L） |  | 0.2±0.24 | 0.2±0.19 |
| Eosinophils（％） |  | 2.1±2.35 | 2.2±2.15 |
| Basophil Count（10^9/L） |  | 0.0±0.06 | 0.0±0.02 |
| Basophils（％） |  | 0.5±0.34 | 0.5±0.51 |
| Erythrocyte Count(RBC)（10^9/L） |  | 4.8±9.10 | 4.4±0.68 |
| Hematocrit (HCT) |  | 37.8±7.09 | 39.7±5.67 |
| Hemoglobin(Hb)（g/L） |  | 125.1±27.77 | 132.8±20.69 |
| Mean Corpuscular Volume(MCV)（fL） |  | 89.2±10.13 | 91.0±5.79 |
| Mean corpuscular hemoglobin（pg） |  | 31.3±21.25 | 30.4±2.23 |
| Platelet Count（10^9/L） |  | 197.3±95.03 | 184.7±68.31 |
| hs-[CRP](https://baike.baidu.com/item/CRP/5495511?fromModule=lemma_inlink)（mg/L） |  | 23.2±46.71 | 5.8±15.92 |
| High-sensitivity troponin（pg/ml） |  | 12.5±57.10 | 10.3±127.87 |
| Procalcitonin(PCT)（ng/ml） |  | 1.6±8.25 | 0.2±0.61 |
| Alpha Fetoprotein(AFP)（ng/ml） |  | 2.4±1.12 | 2.9±1.6 |
| Carcinoembryonic Antigen(CEA)（ng/ml） |  | 3.5±3.25 | 2.8±1.68 |
| Ferritin（ng/ml） |  | 287.7±263.44 | 251.6±244.16 |
| AST(U/L) |  | 31.2±17.69 | 27.3±13.22 |
| ALT(U/L) |  | 27.9±27.06 | 27.3±22.82 |
| TBil (μmoI/L) |  | 13.9±6.72 | 15.6±6.93 |
| eGFR（ml/min） |  | 73.6±30.59 | 86.6±23.86 |
| Urea（mmol/L） |  | 8.0±5.04 | 6.6±4.03 |
| Uric Acid (UA)(mmol/L) |  | 356.9±119.88 | 335.2±91.68 |
| Creatinine(μmoI/L) |  | 106.9±111.42 | 77.3±61.43 |
| Urea to Creatinine ratio(U/C) |  | 87.0±34.08 | 92.3±31.22 |
| TC (mmol/L) |  | 4.6±1.82 | 4.3±1.06 |
| TG (mmol/L) |  | 2.2±3.09 | 1.9±1.58 |

BMI, Body Mass Index; DBP, Diastolic Blood Pressure; SBP, Systolic Blood Pressure; TZD, Thiazolidinedione; DPP-4, Dipeptidyl-peptidase-4; SGLT-2, Sodium-Glucose Transport Protein 2; GLP-1, Glucagon-Like Peptide-1; ACEI, Angiotension Converting Enzyme inhibitors; ARB, Angiotensin receptor blocker; NASIDs, Nonsteroidal Antiinflammatory Drugs; HbAlc, HemoglobinA1c; RBC, Red Blood Cell; WBC, White Blood Cell; AST, Aspartate aminotransferase; ALT, Alanine aminotransferase; TBiL, Total bilirubin; eGFR, estimated Glomerular Filtration Rate; TC, Total cholesterol; TG, Triglyeride.

Table S3 Variable assignment

| **Number** | **Variable** | **Assignment (Reference range)** |
| --- | --- | --- |
| Y | UTIs | 1, Yes; 0, No |
| X1 | Gender | 1, Male; 0, Female |
| X2 | Menopausal or not | 1, Yes; 0, No |
| X3 | Age | Metric variable |
| X4 | Marital status | 1, Married; 2, Unmarried |
| X5 | Length of Stay | Metric variable |
| X6 | Diabetes duration | Metric variable |
| X7 | History of UTIs | 1, Yes; 0, No |
| X8 | History of invasive procedure | 1, Yes; 0, No |
| X9 | BMI | 20~25 kg/m^2^ |
| X10 | Smoking history | 1, Yes; 0, No |
| X11 | Drinking history | 1, Yes; 0, No |
| X12 | DBP | 60~89 mmHg |
| X13 | SBP | 90~139 mmHg |
| X14 | Hypertension | 1, Yes; 0, No |
| X15 | Arrhythmias | 1, Yes; 0, No |
| X16 | Heart Failure | 1, Yes; 0, No |
| X17 | Diabetic peripheral vascular diseases | 1, Yes; 0, No |
| X18 | Coronary Artery Disease | 1, Yes; 0, No |
| X19 | Other Cardiovascular Diseases | 1, Yes; 0, No |
| X20 | Diabetic Nephropathies | 1, Yes; 0, No |
| X21 | Nephrosis | 1, Yes; 0, No |
| X22 | Kidney Calculi | 1, Yes; 0, No |
| X23 | Urinary Calculi | 1, Yes; 0, No |
| X24 | Urinary Incontinence | 1, Yes; 0, No |
| X25 | Other Urologic Diseases | 1, Yes; 0, No |
| X26 | Diabetic Retinopathy | 1, Yes; 0, No |
| X27 | Other Eye Diseases | 1, Yes; 0, No |
| X28 | Diabetic peripheral neuropathy | 1, Yes; 0, No |
| X29 | Other nervous System Diseases | 1, Yes; 0, No |
| X30 | Liver Diseases | 1, Yes; 0, No |
| X31 | Gastrointestinal | 1, Yes; 0, No |
| X32 | Other Digestive System Diseases | 1, Yes; 0, No |
| X33 | Other Urogenital Diseases | 1, Yes; 0, No |
| X34 | Dyslipidemias | 1, Yes; 0, No |
| X35 | Osteoporosis | 1, Yes; 0, No |
| X36 | Hyperuricacidemia | 1, Yes; 0, No |
| X37 | Other Endocrine System Diseases | 1, Yes; 0, No |
| X38 | Dementia | 1, Yes; 0, No |
| X39 | Depression | 1, Yes; 0, No |
| X40 | Other Mental Disorders | 1, Yes; 0, No |
| X41 | Rheumatoid arthritis | 1, Yes; 0, No |
| X42 | Other Immune System Diseases | 1, Yes; 0, No |
| X43 | Anemia | 1, Yes; 0, No |
| X44 | Other Hematologic Diseases | 1, Yes; 0, No |
| X45 | Insulin | 1, taken; 2, not taken |
| X46 | Metformin | 1, taken; 2, not taken |
| X47 | Sulfonylurea | 1, taken; 2, not taken |
| X48 | Glinides | 1, taken; 2, not taken |
| X49 | α-glucosidase inhibitor | 1, taken; 2, not taken |
| X50 | TZD | 1, taken; 2, not taken |
| X51 | DPP-4 inhibitors | 1, taken; 2, not taken |
| X52 | SGLT-2i | 1, taken; 2, not taken |
| X53 | GLP-1 receptor agonist | 1, taken; 2, not taken |
| X54 | Finasteride | 1, taken; 2, not taken |
| X55 | Calcium channel blocker | 1, taken; 2, not taken |
| X56 | ACEI | 1, taken; 2, not taken |
| X57 | ARB | 1, taken; 2, not taken |
| X58 | Diuretics | 1, taken; 2, not taken |
| X59 | β-blocker | 1, taken; 2, not taken |
| X60 | Statins | 1, taken; 2, not taken |
| X61 | NSAIDs | 1, taken; 2, not taken |
| X62 | Fasting plasma glucose | 3.9～6.1 mmol/L |
| X63 | HbA1c | 4%～6% |
| X64 | Microalbuminuria | 0~30mg/L |
| X65 | 24-hour urinary protein quantification | 0.028~0.270 g/24h |
| X66 | Urinary protein | 0:-; 1:±; 2:+; 3:2+; 4:3+; 5:4+ |
| X67 | Urine glucose | 0:-; 1:±; 2:+; 3:2+; 4:3+; 5:4+ |
| X68 | Urine ketones | 0:-; 1:±; 2:+; 3:2+; 4:3+; 5:4+ |
| X69 | Urine occult blood | 0:-; 1:±; 2:+; 3:2+; 4:3+; 5:4+ |
| X70 | Leukocyte esterase | 0:-; 1:±; 2:+; 3:2+; 4:3+; 5:4+ |
| X71 | Urine Leukocyte Counts | 0~5/HP |
| X72 | RBC in Urine | 0~3/HP |
| X73 | Urine epithelial cells counts | 0~5/uL |
| X74 | Leukocyte Count (WBC) | 3.50~9.50 10^9/L |
| X75 | Neutrophil Count | 1.80~6.30 10^9/L |
| X76 | Neutrophils | 40.0~75.0% |
| X77 | Lymphocyte Count | 1.10~3.20 10^9/L |
| X78 | Lymphocytes | 20.0~50.0% |
| X79 | Monocyte Count | 0.10~0.60 10^9/L |
| X80 | Monocytes | 3.0~10.0 % |
| X81 | Eosinophil Count | 0.02~0.52 10^9/L |
| X82 | Eosinophils | 0.4~8.0% |
| X83 | Basophil Count | 0.00~0.06 10^9/L |
| X84 | Basophils | 0.0~1.0% |
| X85 | RBC | 3.80~5.10 10^12/L |
| X86 | HCT | 40.0~50.0 |
| X87 | Hb | 117~150g/L |
| X88 | MCV | 82.0~100.0fL |
| X89 | Mean corpuscular hemoglobin(pg) | 27.0~34.0g/L |
| X90 | Platelet Count | 101~320 10^9/L |
| X91 | hs-[CRP](https://baike.baidu.com/item/CRP/5495511?fromModule=lemma_inlink) | 0.0~5.00mg/L |
| X92 | High-sensitivity troponin | 0.0~9.0ng/L |
| X93 | PCT | 0.0~0.05ng/mL |
| X94 | AFP | 0~7.0ng/mL |
| X95 | CEA | 0~5.0ng/mL |
| X96 | Ferritin | 4.63~204ng/mL |
| X97 | AST | 0～40U/L |
| X98 | ALT | 0～40U/L |
| X99 | TBil | 0.0~21.0umol/L |
| X100 | eGFR | 90~120 ml/（min·1.73m^^2^） |
| X101 | Urea | 2.60~7.50mmol/L |
| X102 | UA | 144~399umol/L |
| X103 | Creatinine | 49.0~82.0umol/L |
| X104 | U/C | 12～20∶1 |
| X105 | TC | 2.83~5.20mmol/L |
| X106 | TG | 0.45～1.69mmol/L |

Table S4 Results of different variable screening methods

| **Methods** | **Included variables** |
| --- | --- |
| No screening | X1 X2 X3 X5 X6 X7 X8 X9 X10 X11 X12 X13 X14 X17 X18 X19 X20 X21 X25 X26 X27 X28 X30 X32 X34 X37 X45 X46 X49 X51 X52 X55 X57 X58 X60 X61 X62 X66 X67 X69 X70 X71 X72 X73 X75 X79 X81 X87 X90 X97 X98 X99 X100 X101 X102 X103 X104 |
| Lasso | X1 X7 X8 X10 X13 X20 X21 X34 X45 X52 X58 X61 X62 X66 X67 X69 X70 X73 X100 X102 X104 X3 X14 X18 X26 X27 X28 X3 X46 X55 X87 X98 X101 X71 X2 X6 X12 X19 X75 X90 X97 X37 X103 X25 X11 X60 X72 X79 X17 X57 |
| Boruta | X1 X5 X7 X8 X13 X45 X52 X58 X62 X66 X69 X70 X71 X72 X73 X75 X87 X99 X100 X101 X102 X103 X104 X79 X9 X3 X6 X9 X27 X61 X90 X25 X81 X18 X2 X60 |

Table S5 The results of internal and external validation of the models

| **Internal /external validation** | **Methods** | **AUC** | | **Accuracy** | | **Precision** | | **Recall** | | **F1 Score** | |
| --- | --- | --- | --- | --- | --- | --- | --- | --- | --- | --- | --- |
|  |  | Mean±SD | 95%CI | Mean±SD | 95%CI | Mean±SD | 95%CI | Mean±SD | 95%CI | Mean±SD | 95%CI |
| **Internal validation** |  |  |  |  |  |  |  |  |  |  |  |
|  | **Data filing** |  |  |  |  |  |  |  |  |  |  |
|  | Modified Random Forest | 0.950±0.033 | 0.949-0.952 | 0.887±0.050 | 0.885-0.889 | 0.903±0.051 | 0.901-0.905 | 0.847±0.104 | 0.843-0.851 | 0.870±0.067 | 0.867-0.872 |
|  | Not | 0.948±0.033 | 0.947-0.950 | 0.886±0.051 | 0.884-0.888 | 0.902±0.059 | 0.900-0.904 | 0.846±0.097 | 0.842-0.850 | 0.869±0.066 | 0.867-0.872 |
|  | Random Forest | 0.951±0.033 | 0.950-0.952 | 0.888±0.049 | 0.886-0.890 | 0.903±0.052 | 0.901-0.905 | 0.848±0.104 | 0.844-0.852 | 0.870±0.067 | 0.868-0.873 |
|  | Simple | 0.951±0.034 | 0.949-0.952 | 0.888±0.050 | 0.886-0.890 | 0.902±0.051 | 0.900-0.905 | 0.849±0.103 | 0.845-0.853 | 0.871±0.067 | 0.868-0.873 |
|  | P value | P=0.0013 | | P=0.3709 | | P=0.7389 | | P=0.0140 | | P=0.2985 | |
|  | **Data balancing** |  |  |  |  |  |  |  |  |  |  |
|  | Borderline SMOTE | 0.940±0.035 | 0.939-0.942 | 0.870±0.057 | 0.868-0.873 | 0.884±0.048 | 0.882-0.887 | 0.854±0.107 | 0.849-0.858 | 0.865±0.072 | 0.862-0.868 |
|  | Random Over Sampler | 0.963±0.029 | 0.962-0.965 | 0.908±0.045 | 0.906-0.910 | 0.924±0.042 | 0.923-0.926 | 0.890±0.076 | 0.887-0.894 | 0.905±0.048 | 0.903-0.907 |
|  | Random Under Sampler | 0.948±0.034 | 0.947-0.950 | 0.880±0.050 | 0.878-0.882 | 0.910±0.055 | 0.908-0.912 | 0.848±0.095 | 0.844-0.852 | 0.874±0.059 | 0.871-0.877 |
|  | SMOTE | 0.947±0.030 | 0.946-0.948 | 0.879±0.049 | 0.877-0.881 | 0.899±0.044 | 0.897-0.901 | 0.857±0.091 | 0.853-0.861 | 0.874±0.059 | 0.872-0.877 |
|  | P value | P<0.0001 | | P<0.0001 | | P<0.0001 | | P<0.0001s | | P<0.0001 | |
|  | **Feature Screening** |  |  |  |  |  |  |  |  |  |  |
|  | Boruta | 0.949±0.034 | 0.948-0.950 | 0.886±0.050 | 0.884-0.888 | 0.900±0.054 | 0.898-0.902 | 0.848±0.101 | 0.844-0.851 | 0.869±0.066 | 0.867-0.871 |
|  | Lasso | 0.952±0.031 | 0.951-0.953 | 0.890±0.048 | 0.888-0.892 | 0.904±0.053 | 0.902-0.906 | 0.852±0.098 | 0.848-0.855 | 0.873±0.063 | 0.871-0.875 |
|  | Not | 0.949±0.035 | 0.948-0.950 | 0.886±0.052 | 0.884-0.888 | 0.904±0.053 | 0.902-0.906 | 0.843±0.106 | 0.839-0.846 | 0.868±0.070 | 0.866-0.870 |
|  | P value | P<0.0001 | | P=0.0174 | | P=0.0011 | | P=0.0056 | | P=0.0237 | |
|  | **Algorithms** |  |  |  |  |  |  |  |  |  |  |
|  | AdaBoost | 0.969±0.015 | 0.968-0.971 | 0.910±0.027 | 0.908-0.912 | 0.911±0.038 | 0.908-0.914 | 0.891±0.059 | 0.886-0.896 | 0.899±0.037 | 0.896-0.902 |
|  | Bagging | 0.967±0.020 | 0.966-0.969 | 0.918±0.035 | 0.916-0.921 | 0.907±0.040 | 0.903-0.910 | 0.913±0.067 | 0.908-0.918 | 0.909±0.045 | 0.905-0.912 |
|  | Bernoulli Naïve Bayes | 0.956±0.023 | 0.954-0.958 | 0.892±0.036 | 0.889-0.895 | 0.896±0.047 | 0.892-0.901 | 0.866±0.059 | 0.861-0.871 | 0.880±0.042 | 0.876-0.883 |
|  | Decision Tree | 0.921±0.032 | 0.919-0.924 | 0.875±0.038 | 0.872-0.878 | 0.872±0.052 | 0.867-0.876 | 0.858±0.075 | 0.852-0.864 | 0.862±0.046 | 0.858-0.866 |
|  | Extra Tree | 0.910±0.039 | 0.907-0.914 | 0.861±0.044 | 0.858-0.865 | 0.866±0.059 | 0.861-0.870 | 0.826±0.094 | 0.818-0.833 | 0.842±0.061 | 0.837-0.847 |
|  | Gaussian Naïve Bayes | 0.939±0.029 | 0.937-0.942 | 0.849±0.069 | 0.844-0.855 | 0.901±0.054 | 0.896-0.905 | 0.762±0.141 | 0.751-0.773 | 0.817±0.095 | 0.810-0.825 |
|  | Gradient Boosting | 0.973±0.014 | 0.972-0.974 | 0.919±0.028 | 0.917-0.922 | 0.921±0.034 | 0.918-0.923 | 0.901±0.062 | 0.896-0.906 | 0.909±0.038 | 0.906-0.912 |
|  | KNN | 0.955±0.022 | 0.954-0.957 | 0.865±0.045 | 0.862-0.869 | 0.943±0.046 | 0.939-0.946 | 0.750±0.151 | 0.738-0.762 | 0.823±0.094 | 0.816-0.831 |
|  | LDA | 0.950±0.024 | 0.948-0.952 | 0.881±0.035 | 0.878-0.883 | 0.915±0.045 | 0.911-0.919 | 0.817±0.070 | 0.812-0.823 | 0.861±0.045 | 0.857-0.865 |
|  | Logistic Regression | 0.953±0.022 | 0.952-0.955 | 0.891±0.033 | 0.888-0.894 | 0.906±0.044 | 0.902-0.909 | 0.853±0.058 | 0.849-0.858 | 0.877±0.039 | 0.874-0.880 |
|  | Multinomial Naïve Bayes | 0.916±0.034 | 0.913-0.919 | 0.837±0.056 | 0.832-0.842 | 0.888±0.057 | 0.883-0.892 | 0.742±0.110 | 0.733-0.752 | 0.803±0.077 | 0.797-0.810 |
|  | Passive Aggressive | 0.912±0.040 | 0.909-0.915 | 0.841±0.051 | 0.837-0.845 | 0.833±0.069 | 0.827-0.838 | 0.828±0.074 | 0.822-0.834 | 0.827±0.055 | 0.823-0.832 |
|  | QDA | 0.950±0.022 | 0.948-0.951 | 0.876±0.042 | 0.872-0.879 | 0.921±0.040 | 0.917-0.924 | 0.802±0.076 | 0.796-0.808 | 0.855±0.050 | 0.851-0.859 |
|  | Random Forest | 0.977±0.017 | 0.976-0.979 | 0.931±0.030 | 0.929-0.934 | 0.921±0.036 | 0.918-0.924 | 0.926±0.061 | 0.921-0.931 | 0.922±0.040 | 0.919-0.925 |
|  | SGD | 0.952±0.022 | 0.950-0.954 | 0.888±0.033 | 0.885-0.890 | 0.906±0.047 | 0.902-0.910 | 0.845±0.077 | 0.838-0.851 | 0.871±0.047 | 0.868-0.875 |
|  | SVM | 0.967±0.020 | 0.965-0.968 | 0.916±0.032 | 0.913-0.918 | 0.915±0.039 | 0.912-0.919 | 0.897±0.061 | 0.892-0.902 | 0.905±0.042 | 0.902-0.909 |
|  | XGBoost | 0.979±0.016 | 0.978-0.980 | 0.932±0.031 | 0.929-0.934 | 0.924±0.037 | 0.921-0.927 | 0.924±0.064 | 0.919-0.929 | 0.923±0.041 | 0.919-0.926 |
|  | P value | P<0.0001 | | P<0.0001 | | P<0.0001 | | P<0.0001 | | P<0.0001 | |
| **External validation** |  |  |  |  |  |  |  |  |  |  |  |
|  | **Data filing** |  |  |  |  |  |  |  |  |  |  |
|  | ModifiedRandomForest | 0.922±0.041 | 0.922-0.923 | 0.874±0.038 | 0.874-0.874 | 0.844±0.075 | 0.844-0.844 | 0.789±0.074 | 0.789-0.789 | 0.812±0.055 | 0.812-0.812 |
|  | Not | 0.939±0.035 | 0.938-0.939 | 0.885±0.039 | 0.885-0.885 | 0.820±0.085 | 0.820-0.820 | 0.818±0.081 | 0.817-0.818 | 0.814±0.060 | 0.814-0.814 |
|  | Random Forest | 0.926±0.038 | 0.926-0.926 | 0.876±0.034 | 0.876-0.876 | 0.846±0.069 | 0.846-0.846 | 0.793±0.077 | 0.793-0.793 | 0.815±0.052 | 0.815-0.815 |
|  | Simple | 0.923±0.041 | 0.923-0.923 | 0.873±0.039 | 0.873-0.873 | 0.844±0.075 | 0.844-0.844 | 0.786±0.077 | 0.786-0.787 | 0.810±0.057 | 0.810-0.811 |
|  | P value | P<0.0001 | | P<0.0001 | | P<0.0001 | | P<0.0001 | | P<0.0001 | |
|  | **Data balancing** |  |  |  |  |  |  |  |  |  |  |
|  | Borderline SMOTE | 0.909±0.045 | 0.909-0.909 | 0.853±0.045 | 0.853-0.853 | 0.786±0.083 | 0.786-0.786 | 0.787±0.070 | 0.787-0.788 | 0.783±0.061 | 0.783-0.784 |
|  | Random Over Sampler | 0.930±0.041 | 0.930-0.930 | 0.886±0.032 | 0.886-0.886 | 0.852±0.060 | 0.852-0.852 | 0.804±0.071 | 0.803-0.804 | 0.824±0.051 | 0.824-0.825 |
|  | Random Under Sampler | 0.938±0.031 | 0.938-0.938 | 0.884±0.029 | 0.884-0.884 | 0.845±0.064 | 0.845-0.845 | 0.812±0.084 | 0.812-0.812 | 0.824±0.047 | 0.823-0.824 |
|  | SMOTE | 0.924±0.038 | 0.924-0.924 | 0.873±0.040 | 0.873-0.873 | 0.813±0.075 | 0.813-0.814 | 0.816±0.067 | 0.816-0.816 | 0.812±0.055 | 0.812-0.812 |
|  | P value | P<0.0001 | | P<0.0001 | | P<0.0001 | | P<0.0001 | | P<0.0001 | |
|  | **Feature Screening** |  |  |  |  |  |  |  |  |  |  |
|  | Boruta | 0.930±0.037 | 0.930-0.930 | 0.876±0.042 | 0.876-0.876 | 0.835±0.084 | 0.835-0.835 | 0.800±0.077 | 0.800-0.800 | 0.813±0.060 | 0.813-0.813 |
|  | Lasso | 0.926±0.040 | 0.926-0.926 | 0.877±0.037 | 0.877-0.877 | 0.838±0.075 | 0.838-0.838 | 0.796±0.078 | 0.795-0.796 | 0.812±0.055 | 0.812-0.812 |
|  | Not | 0.926±0.040 | 0.926-0.927 | 0.878±0.035 | 0.878-0.878 | 0.842±0.072 | 0.842-0.842 | 0.794±0.080 | 0.794-0.795 | 0.813±0.053 | 0.813-0.814 |
|  | P value | P<0.0001 | | P<0.0001 | | P<0.0001 | | P<0.0001 | | P<0.0001 | |
|  | **Algorithms** |  |  |  |  |  |  |  |  |  |  |
|  | AdaBoost | 0.959±0.016 | 0.959-0.959 | 0.898±0.025 | 0.898-0.898 | 0.855±0.052 | 0.855-0.856 | 0.842±0.055 | 0.842-0.842 | 0.847±0.037 | 0.847-0.847 |
|  | Bagging | 0.953±0.018 | 0.953-0.953 | 0.899±0.028 | 0.898-0.899 | 0.852±0.056 | 0.852-0.853 | 0.849±0.051 | 0.848-0.849 | 0.849±0.041 | 0.849-0.849 |
|  | Bernoulli Naïve Bayes | 0.939±0.019 | 0.939-0.939 | 0.879±0.027 | 0.878-0.879 | 0.820±0.058 | 0.819-0.820 | 0.823±0.051 | 0.822-0.823 | 0.819±0.038 | 0.819-0.819 |
|  | Decision Tree | 0.905±0.033 | 0.905-0.905 | 0.867±0.037 | 0.867-0.867 | 0.797±0.081 | 0.796-0.797 | 0.828±0.068 | 0.827-0.828 | 0.808±0.049 | 0.808-0.808 |
|  | Extra Tree | 0.870±0.045 | 0.870-0.871 | 0.840±0.043 | 0.840-0.841 | 0.764±0.088 | 0.764-0.765 | 0.778±0.081 | 0.777-0.778 | 0.766±0.060 | 0.766-0.766 |
|  | Gaussian Naïve Bayes | 0.933±0.030 | 0.933-0.933 | 0.882±0.024 | 0.882-0.882 | 0.855±0.053 | 0.855-0.855 | 0.787±0.061 | 0.786-0.787 | 0.817±0.037 | 0.817-0.817 |
|  | Gradient Boosting | 0.962±0.016 | 0.962-0.962 | 0.905±0.023 | 0.905-0.905 | 0.874±0.048 | 0.874-0.874 | 0.843±0.055 | 0.842-0.843 | 0.856±0.035 | 0.856-0.857 |
|  | KNN | 0.916±0.028 | 0.916-0.916 | 0.857±0.028 | 0.857-0.857 | 0.867±0.080 | 0.866-0.867 | 0.696±0.100 | 0.695-0.696 | 0.763±0.056 | 0.763-0.764 |
|  | LDA | 0.924±0.026 | 0.923-0.924 | 0.871±0.029 | 0.871-0.871 | 0.842±0.068 | 0.842-0.843 | 0.765±0.060 | 0.765-0.765 | 0.799±0.043 | 0.799-0.799 |
|  | Logistic Regression | 0.926±0.025 | 0.926-0.926 | 0.876±0.028 | 0.876-0.876 | 0.832±0.063 | 0.832-0.833 | 0.798±0.056 | 0.798-0.798 | 0.813±0.042 | 0.812-0.813 |
|  | Multinomial Naïve Bayes | 0.880±0.035 | 0.880-0.880 | 0.848±0.039 | 0.848-0.848 | 0.842±0.097 | 0.842-0.843 | 0.692±0.064 | 0.692-0.692 | 0.754±0.049 | 0.754-0.754 |
|  | Passive Aggressive | 0.885±0.048 | 0.884-0.885 | 0.831±0.050 | 0.831-0.831 | 0.746±0.097 | 0.745-0.746 | 0.777±0.071 | 0.776-0.777 | 0.756±0.064 | 0.756-0.757 |
|  | QDA | 0.930±0.027 | 0.930-0.930 | 0.886±0.025 | 0.886-0.886 | 0.873±0.054 | 0.873-0.873 | 0.777±0.059 | 0.777-0.778 | 0.820±0.039 | 0.820-0.820 |
|  | Random Forest | 0.962±0.014 | 0.962-0.962 | 0.911±0.021 | 0.911-0.911 | 0.881±0.046 | 0.881-0.881 | 0.853±0.050 | 0.853-0.853 | 0.865±0.033 | 0.865-0.865 |
|  | SGD | 0.923±0.026 | 0.923-0.923 | 0.871±0.033 | 0.871-0.872 | 0.827±0.076 | 0.827-0.827 | 0.793±0.067 | 0.792-0.793 | 0.806±0.046 | 0.805-0.806 |
|  | SVM | 0.943±0.018 | 0.943-0.943 | 0.891±0.022 | 0.891-0.892 | 0.856±0.048 | 0.855-0.856 | 0.818±0.056 | 0.818-0.818 | 0.835±0.034 | 0.834-0.835 |
|  | XGBoost | 0.956±0.015 | 0.956-0.956 | 0.897±0.022 | 0.897-0.897 | 0.869±0.048 | 0.869-0.869 | 0.820±0.050 | 0.820-0.820 | 0.842±0.035 | 0.842-0.843 |
|  | P value | P<0.0001 | | P<0.0001 | | P<0.0001 | | P<0.0001 | | P<0.0001 | |
